# Supplementary material for: Using Detached Industrial Hemp Leaf Inoculation Assays to Screen for Varietal Susceptibility and Product Efficacy on Botrytis cinerea
Source: Plants (Basel). 2023 Sep 15;12(18):3278. doi: 10.3390/plants12183278 (PMC10536681; doi:10.3390/plants12183278)
Supplement: Supplementary file 1 [file plants-12-03278-s001.zip › plants-2595122-supplementary.pdf]

Supplementary Materials

Table S1. Average daily lesion length results from four repeated experiments (Exp A & B) screening the efficacy of products against *B. cinerea.* Different letters represent significant difference between treated and untreated within experiments.

	Ave daily lesion length (mm)							
Product	Exp 1A	Exp 1B	Exp 2A	Exp 2B	Exp 3A	Exp 3B	Exp 4A	Exp 4B
5	3.75a	1.18a						
4	3.80a	1.13a						
3	7.40b	6.25b						
2	7.45b	6.92b						
1	7.72b	-						
9			1.19a	4.05a				
8			2.21a	6.06a				
6			7.72b	8.15b				
7			11.29c	8.37b				
14					1.05a	0.88a		
13					4.20ab	1.68a		
12					6.38bc	7.97cd		
11					6.59bcd	6.37bc		
10					8.00cd	4.72b		
16							Phytotoxic	Phytotoxic
17							2.77a	4.83a
20							5.78b	6.90bc
18							6.29bc	5.71ab
19							7.89cd	8.33cd
21							8.16cd	8.42cd
15							8.69d	8.18cd
22							9.50d	9.24d
Untreated Control (*B. cinerea* plug)	8.27b	6.53b	12.17c	8.76b	9.76d	9.43d	8.77d	9.12d
p-value	0.018	<0.001	<0.001	<0.001	<0.001	<0.001	<0.001	<0.001
LSD	3.252	1.660	2.308	2.26	2.132	1.918	1.199	1.016
Replicates (n)	5	20	20	20	20	20	20	20

Table S2. Detached leaf assay results for four repeated experiments screening the efficacy of products with amended pH against *B. cinerea.* Different letters represent significant difference between treated and untreated within experiments.

Treatment	Ave daily lesion length (mm)							
Product	Exp 5A	Exp 5B	Exp 6A	Exp 6B	Exp 7A	Exp 7B	Exp 8A	Exp 8B
Treatment 14 pH3	7.71cd	6.00cd						
Treatment 14 pH4	4.51abc	3.59abc						
Treatment 14 pH5	8.28cd	2.30ab						
Treatment 14 pH6	5.53bc	5.12bcd						
Treatment 14 pH7	5.11bc	8.67de						
Treatment 14 pH8	2.75ab	7.93de						
Treatment 14 pH9	1.01a	3.17abc						
Treatment 14 pH10	4.92abc	1.33a						
Treatment 8 pH3			2.80ab	7.65c				
Treatment 8 pH4			5.30abc	4.73bc				
Treatment 8 pH5			6.30bc	5.35bc				
Treatment 8 pH6			2.07a	4.35abc				
Treatment 8 pH7			4.15abc	3.76ab				
Treatment 8 pH8			5.58bc	3.29ab				
Treatment 8 pH9			4.7abc	4.11ab				
Treatment 8 pH10			4.10abc	1.23a				
Treatment 5 pH3					8.67de	5.28ab		
Treatment 5 pH4					9.01ef	6.76bc		
Treatment 5 pH5					7.72de	3.78ab		
Treatment 5 pH6					5.88cd	5.62abc		
Treatment 5 pH7					2.49ab	3.03a		
Treatment 5 pH8					0.20a	2.39a		
Treatment 5 pH9					0.68ab	3.86ab		
Treatment 5 pH10					3.42bc	2.55a		
Treatment 9 pH3							0.00a	0.00a
Treatment 9 pH4							0.00a	0.00a
Treatment 9 pH5							0.00a	0.00a
Treatment 9 pH6							2.88b	0.97a
Treatment 9 pH7							1.36ab	5.22b
Treatment 9 pH8							0.63a	0.00a
Treatment 9 pH9							0.93ab	0.00a
Treatment 9 pH10							1.06ab	0.00a
Untreated Control (*B. cinerea* plug)	10.94d	11.48e	10.94d	11.48d	11.89f	8.53c	11.89c	8.53c
p-value	<0.001	<0.001	<0.001	<0.001	<0.001	<0.001	<0.001	<0.001
LSD	2.455	2.209	1.996	2.03	1.769	1.961	1.367	1.349
Replicates (n)	20	20	20	20	20	20	20	20
